# A rare skin infection in atopic dermatitis: A case report

**DOI:** 10.1002/ccr3.3167

**Published:** 2020-09-14

**Authors:** Giovanna Malara, Caterina Trifirò, Annunziata Bartolotta, Marco Conte, Pietro Denisi

**Affiliations:** ^1^ Dermatology Department Grande Ospedale Metropolitano Bianchi‐Melacrino‐Morelli Reggio Calabria Italy; ^2^ Microbiology Department Grande Ospedale Metropolitano Bianchi‐Melacrino‐Morelli Reggio Calabria Italy; ^3^ AGRARIA Department Mediterranea University of Reggio Calabria Reggio Calabria Italy

**Keywords:** atopic dermatitis, *Enterococcus faecalis*, skin infection, *Staphylococcus aureus*

## Abstract

Atopic dermatitis is associated with a susceptibility to infection usually by Staphylococcus spp due to a decrease of AMPs and Th2 cytokines (eg, IL‐17). We reported a rare E. faecalis skin contamination in AD patients due to a frequent contact with excrement.

## Graphical abstract

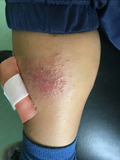

## 1 INTRODUCTION

Atopic dermatitis (AD) pathophysiology is complex and results from skin barrier dysfunction and an unregulated immune response, and this condition is influenced by genetic and environmental factors.^1^ Patients with AD are also at increased risk of bacterial, fungal, and viral skin infections which can lead to invasive infections if left untreated.^2^, ^3^ Furthermore, their greater predisposition to developing skin infections would seem to be related to various contributing factors including skin barrier defects, a decrease in antimicrobial peptides (AMPs), an increased skin pH, or Th2 cytokines (such as IL‐4 and IL‐13).^3^

## 2 CASE REPORT

**FIGURE 1:**
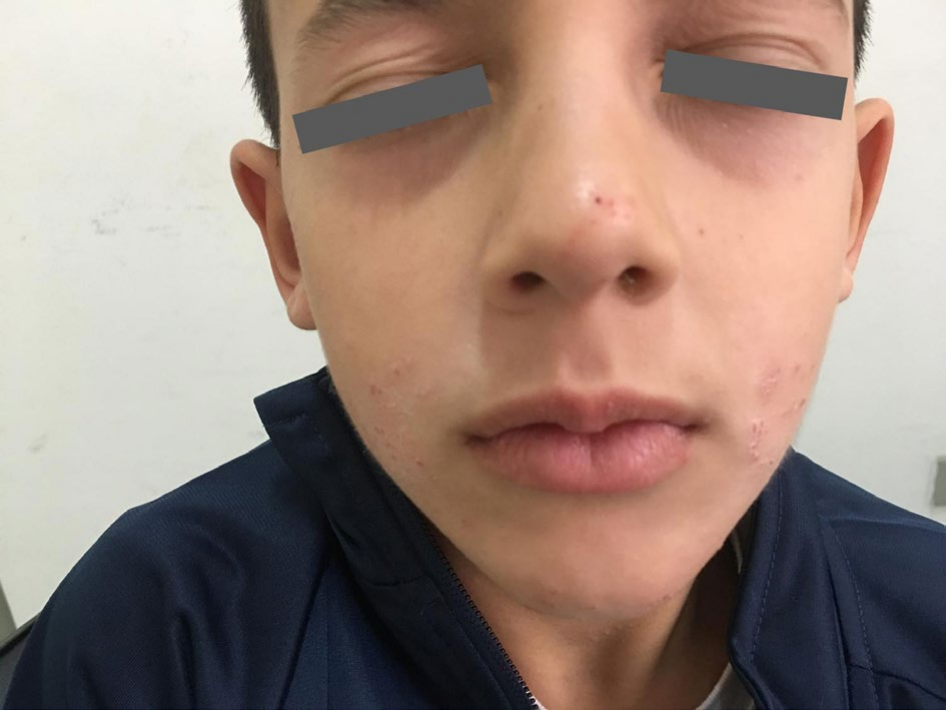
AD in a 9‐y‐old boy, crusted lesions on the face

**FIGURE 2:**
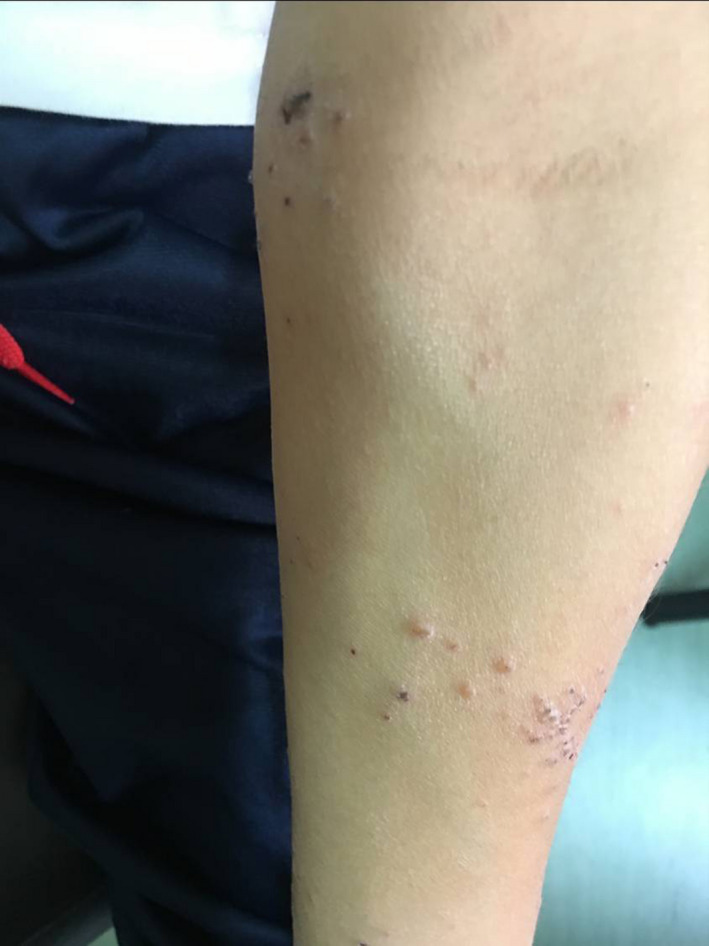
AD in a 9‐y‐old boy, crusted lesions on the lower limb

**FIGURE 3:**
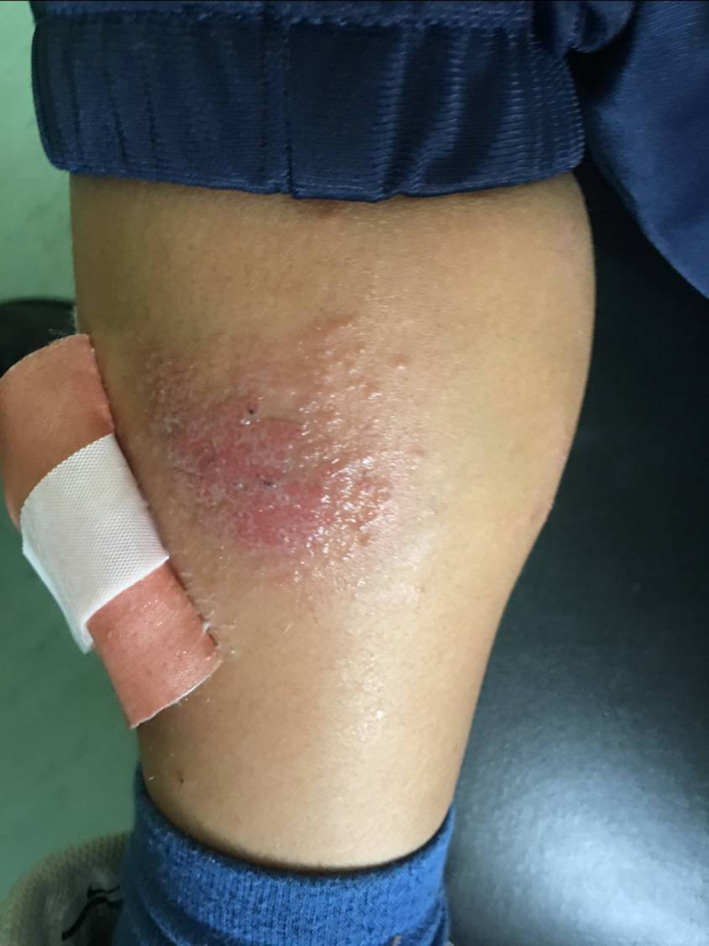
Serious itchy plaque on the lower left limb in a 9‐y‐old boy suffering from AD

A 9‐year‐old Caucasian boy suffering from chronic AD since the age of 1 year, who had spent a lot of time at a horse riding center, visited the clinic in January 2019. He reported worsening pruritus and scabs on his face, trunk, and extremities along with a diffused skin dryness (Figures 1 and 2) and a very serious itchy plaque on his lower left limb (Figure 3). As no laboratory test is needed to identify atopic dermatitis, the diagnosis was made by examining the skin and reviewing the boy's medical history. The patient had already undergone skin prick tests for respiratory or food allergies, and patch tests for contact dermatitis, all of which resulted negative. In addition, the serum level of total immunoglobulin E was measured and was found to be within the normal range. He was treated for a long time with both topical steroids (including hydrocortisone butyrate 0.1%, mometasone furoate 0.1%) and inhibitors of calcineurin (tacrolimus and pimecrolimus) with minimal benefit. Afterward, he was treated with deflazacort oral solution at a dosage of 1 mg/kg/die for 20 days in several cycles. Despite the treatment, his disease often flared up again.

When he came for his first checkup, the patient had visible Dennie‐Morgan lines, inflamed, erythematous papules, and crusty plaques scattered over 75% of his face and body, resulting in an eczema area and severity index (EASI) score of 19.

He also had a reddish plaque on his left leg, which was resistant to both steroidal and antimicrobial topical treatments. We therefore decided to perform a culture test of a skin scraped specimen which revealed an *Enterococcus faecalis* (*E. faecalis*) on the lesion.

An antibiogram test allowed us to choose the most effective antibiotic drug. This subsequently led to a rapid improvement of the infected skin lesion. The patient had spent a substantial period of time at the horse riding center and had therefore came into frequent contact with horses and their excrement.

Wolny‐Koładka (2018) and Graves et al.(2008) identify some species *Staphylococcus* and *Enterococcus* spp. from the ground of a horse riding center. This all confirmed the high probability of contracting Staphylococcal and Enterococcal when exposed to soil contaminated with horse feces, as in the case of our patient.^4^, ^5^

According to recent insight on physiopathology, it is well known that AD is associated with decreased production of AMPs in the skin, which are a group of molecules which act as protection against bacteria, fungi, and viruses.^6^, ^7^ The reduction might be due to the suppressive effect of Th2 cytokines (present in higher quantities in AD) and a relative decrease in IL‐17 (an inducer of AMPs).^3^, ^8^

Among skin infections, the most common bacterial skin infections in AD are caused by *Staphylococcus aureus* (*S aureus*) followed by *Streptococcus pyogenes* whose virulence is due to staphylococcal enterotoxins (superantigens). More than 80% of *S aureus* isolated from AD patients are superantigen‐producing.^9^

Patients with AD are at increased risk of colonization by methicillin‐resistant *S. aureus* (MRSA), compared with the general population.^10^ MRSA skin and soft‐tissue infections lead to a loop of AD.^11^

Like bacterial infections, AD patients are at a higher risk of eczema herpeticum (EH) caused by the herpes simplex virus and fungal infection, such as tinea or yeast.^12^

## 3 CONCLUSION

Skin barrier defects, a decrease in AMPs, increased skin pH, or Th2 cytokines are potential contributing factors to the amplified risk of skin infections in AD, especially those caused by *S. aureus*. Conversely, our patient developed a skin infection caused by *E faecalis,* which is a bacterium typically present in the gut and bowel. The combination of environmental factors (such as frequent contact with animal excrement) and a well‐documented higher susceptibility of AD patients to skin infections go some way to explaining this unusual skin condition.

## CONFLICT OF INTEREST

Authors have no conflict of interest.

## AUTHOR CONTRIBUTION

MG: conceived the article and wrote the paper. TC, BA, and CM: critically revised the manuscript. DP: gave important intellectual contribution to the final version of the article.

## CONSENT STATEMENT

A written informed consent was obtained from the patient.
